# Home Oxygen Therapy-Induced Bladder Rupture

**DOI:** 10.7759/cureus.16975

**Published:** 2021-08-07

**Authors:** Shoryu Takayama, Kohei Takura

**Affiliations:** 1 Surgery, Nagoya Tokushukai General Hospital, Nagoya, JPN

**Keywords:** home oxygen therapy, urinary catheter, bladder rupture, abdominal compartment syndrome, medical accident, home medical care

## Abstract

An 84-year-old man taking home oxygen therapy (HOT) for chronic obstructive pulmonary disease (COPD) was brought to our emergency department because of cardiopulmonary arrest after connecting an oxygen cylinder to a urinary catheter at home. On physical examination, subcutaneous emphysema and abdominal distension were noted. The oxygen stored in the abdominal cavity seemed to induce abdominal compartment syndrome, so we decided to drain the oxygen. Advanced cardiac life support protocol and drainage were performed, followed by the return of spontaneous circulation. The number of patients on HOT for COPD is expected to increase because COPD is a common disease globally. This patient had a urinary catheter due to urinary retention caused by benign prostatic hyperplasia (BPH). BPH is a benign tumor of the prostate gland that is the most common cause of dysuria in older men. COPD and BPH are very common diseases, so similar medical accidents may occur. We report this case to prevent a recurrence.

## Introduction

In this report, we describe a case of death due to bladder rupture caused by an incorrect connection of an oxygen cylinder for home oxygen therapy (HOT) to a urinary catheter. Although the safety of medical equipment has been improving year by year and the number of medical accidents seems to be decreasing, medical accidents are still reported. Some case reports have described such medical accidents. For example, a case of tension pneumothorax caused by the incorrect connection of an intubation tube to the oxygen supply tube has been reported [1].

Chronic obstructive pulmonary disease (COPD) is a common disease globally. The prevalence of COPD was reported to range from 7.8% to 19.4% in Latin American cities in the PLATINO study [2]. In the BOLD Study, the prevalence of COPD with Global Initiative for Chronic Obstructive Lung Disease stage 2 or higher in Western countries aged 40 years or older were reported to be 16.4% in males, 8.5% in females, and 10.4% overall in Western countries [3]. COPD is a common disease worldwide. In addition, the usefulness of HOT for COPD has been known. Therefore, the number of HOT patients is expected to increase worldwide. Our patient had a urinary catheter due to urinary retention caused by benign prostatic hyperplasia (BPH). BPH is a benign tumor of the prostate gland that is the most common cause of dysuria in older men. Histologically, BPH is present in more than 50% of men aged 60 years old. In addition, approximately, 90% of men are expected to have BPH until the age of 85 years old, and one-fourth of them are expected to have clinical symptoms [4]. As the prevalence of BPH is increasing, the number of patients using a urinary catheter will increase. We report this case for the prevention of bladder rupture similar to this patient in the future.

## Case presentation

**Figure 1:**
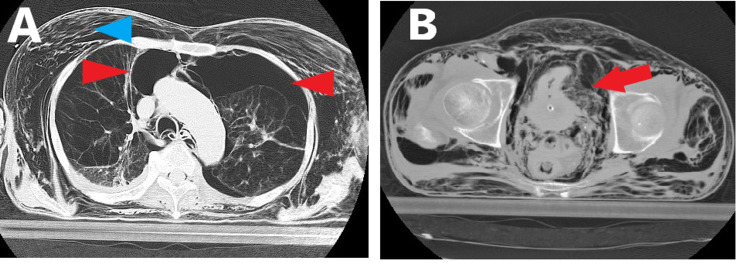
CT scan after the ROSC. CT showed massive retroperitoneal emphysema, subcutaneous emphysema (A, blue arrowhead), intra-abdominal air, and bilateral pneumothorax (A, red arrowhead). Although it was difficult to identify the site of injury due to intra-abdominal air and retroperitoneal emphysema, the diagnosis of bladder rupture was made due to the irregularity of the bladder wall (B, red arrow). CT: computer tomography; ROSC: return of spontaneous circulation

An 84-year-old man taking HOT for COPD was brought to our emergency department (ED) because of cardiopulmonary arrest after connecting an oxygen cylinder to a urinary catheter at home. The initial electrocardiogram (ECG) showed asystole. He had erroneously connected an oxygen cylinder to his urinary catheter at home. He screamed loudly in pain, and his wife noticed something happened. He had been suffering from intractable urinary retention due to BPH. Medication did not improve his urinary retention symptoms, and he had a urinary catheter placed for the first time a few days before he was rushed to the ED. His wife stated that he was used to handling an oxygen cylinder, but not a urinary catheter yet. His wife disconnected the oxygen cylinder and urinary catheter and called for an ambulance. His physical examination revealed subcutaneous emphysema from the face to the legs and abdominal distension. It was presumed that oxygen was stored in the abdominal cavity and subcutaneous space. The urinary catheter was not connected to a urinary bag. We suggested that he had incorrectly connected the urinary catheter’s connector for a urinary bag to the home oxygen cylinder. The advanced cardiac life support (ACLS) protocol was continued but the return of spontaneous circulation (ROSC) was not achieved. The oxygen stored in the abdominal cavity seemed to induce abdominal compartment syndrome (ACS), so we decided to drain the oxygen. An umbilical incision was made and the peritoneum was carefully split with Pean forceps. The oxygen in the abdominal cavity was drained. After drainage, the ECG changed to pulseless electrical activity, and ACLS protocol was continued, following which ROSC was achieved. The pupils were dilated and the pupillary light reflex disappeared after ROSC. The neurological prognosis was predicted to be poor. His blood test results showed a white blood cell count of 116 × 102/μl, hemoglobin 9.9 g/dL, platelet 21.2 × 104/μL, creatine kinase 76 U/L, aspartate aminotransferase 383 U/L, alanine aminotransferase 270 U/L, amylase 191 U/L, total protein 5.0 g/dL, albumin 2.3 g/dL, creatinine 1.49 mg/dL, blood urea nitrogen 26.9 mg/dL, sodium 152 mEq/L, potassium 4.5 mEq/L, chlorine 95 mEq/L, blood sugar 229 mg/dL, C-reactive protein 2.83 mg/dL, prothrombin time-international normalized ratio 1.69, and activated partial thromboplastin time 35.5 seconds. The patient was intubated and a computed tomography (CT) scan was performed under artificial respiration. CT showed massive retroperitoneal emphysema, subcutaneous emphysema, intra-abdominal air, and bilateral pneumothorax (Figure 1A). Although it was difficult to identify the site of injury due to intra-abdominal air and retroperitoneal emphysema, a diagnosis of bladder rupture was made due to the irregularity of the bladder wall (Figure 1B) and the medical history. CT showed that the balloon of the urinary catheter was functioning, so it was considered that he had incorrectly connected the urinary catheter’s connector for a urinary bag to the home oxygen cylinder. The patient was admitted to the invasive care unit (ICU) for systemic management. His blood pressure was 86/40 mmHg and his heart rate was 50 beats per minute at admission. Gradually, bradycardia was observed and his condition worsened. When we explained to the patient’s family that the neurological prognosis was poor, they did not wish to continue treatment. The patient died five hours after admission to the ICU. The patient’s family declined an autopsy.

## Discussion

Such a rare case of incorrect connection has not been reported previously. However, COPD and BPH are very common diseases worldwide, and the number of patients with HOT and a urinary catheter will increase. Therefore, it is significant to report this case to prevent the recurrence of similar cases. Although the safety of medical equipment has been improving year by year and the number of medical accidents seems to be decreasing, medical accidents are still reported. A case of tension pneumothorax caused by the incorrect connection of the intubation tube to the oxygen supply tube has been reported [1]. Owing to the improvement in ventilator devices, incorrect connection cases like this are undoubtedly decreasing. Medical accident case reports may reduce the incidence of medical accidents. The connection port of the oxygen cylinder and the urine catheter is easy to connect, and it is necessary to design other connection ports to avoid an incorrect connection. To avoid recurrence like this case, it is important not only to design devices but also to educate patients. When we see patients with HOT and a urinary catheter, we should educate them not to connect HOT and a urinary catheter.

## Conclusions

Although medical accidents seem to be on a gradual downward trend owing to innovations in the design of medical equipment or patient education, they are still reported. We experienced a patient who died after incorrectly connecting an oxygen cylinder to a urinary catheter. Such cases of medical accidents have not been reported. Similar cases may occur in the future because the prevalence of COPD and BPH are increasing worldwide. It is necessary to design other connection ports to avoid an incorrect connection and to educate patients not to make such incorrect connections, although this is a very rare case.
